# Antibacterial and Antidiarrheal Activities of Plant Products against Enterotoxinogenic *Escherichia coli*

**DOI:** 10.3390/toxins5112009

**Published:** 2013-11-07

**Authors:** J. Daniel Dubreuil

**Affiliations:** Département de pathologie et microbiologie, Faculté de médecine vétérinaire, Université de Montréal, Québec, Canada; E-Mail: daniel.dubreuil@umontreal.ca; Tel.: +1-450-773-8521 (ext.8433); Fax: +1-450-778-8108

**Keywords:** enterotoxigenic *Escherichia coli*, plant product, enterotoxin, antibacterial, diarrhea, treatment, traditional medicine, toxin inhibition, secretion, extracts

## Abstract

Enterotoxigenic *Escherichia coli* (ETEC) produces two types of enterotoxins: heat-labile (LT) and heat-stable (STa and STb). These molecules are involved in the induction of secretory diarrhea in animals including humans. This condition is currently treated using a fluid replacement therapy and antibiotics. This treatment is often not available to people in developing countries, and several die from the condition provoke by ETEC. Over the years, plants and plant extracts have been use as traditional medicine to treat various gastrointestinal ailments including diarrhea. Many of these plant products have been claimed to be active against diarrhea, however few have been extensively studied. The main objective of this review was to gather the scattered information on the antidiarrheal activities reported for various plant products on ETEC. This includes two major effects: (1) The inhibitory effect on bacterial growth or viability and (2) The interference with ETEC enterotoxins activity upon the intestinal epithelium. We will focus on plant products and extracts for which we have major indications of their biological activity against ETEC and their enterotoxins. Because *Vibrio cholerae* toxin (CT) is structurally, antigenically and mechanistically related to LT, it will also be discussed in this review.

## 1. Introduction

Plants and their products have been used by humans for treatments of numerous diseases for thousands of years. Traditional medicine (also known as indigenous or folk medicine) comprises knowledge that developed over generations within various societies before the era of modern medicine [1]. The plants that showed healing powers are referred as medicinal. Historically, these treatments would cure or relieve symptoms. However, poisonings also occur at a high rate. Dating back to prehistory, people of all continents have use infusions and poultices probably of thousand plants. Neanderthals, 60,000 years ago, used plants such as hollyhock for disease treatment that are still used today in many countries [2]. For example, Ayurveda, the science of life, a comprehensive medical system described by ancient herbalists almost 6000 years ago is still exploited by local Indian people for treatment of illnesses. In fact, about 80% of the population of the developing countries still uses traditional medicines for their health care [3]. One of the reasons explaining this situation is the cost and limited access to modern therapies. 

In western countries, alternative or complementary medicine refers to traditional medicine used outside its traditional culture. Today, many plant compounds are readily available as over-the-counter self-medication. These preparations are relatively unregulated and as a result, herbal suppliers and natural food stores provide the customers with variable amounts of active substances of more or less controlled purity. 

In recent years, the interest in the study of medicinal plants as a source of pharmacologically active compounds has increased worldwide. Nevertheless, many plants have not been studied yet for the claimed biological activities and possible adverse effects. It is estimated that there is approximately 500,000 species of plants on earth [4]. Only a relatively small percentage, 1% to 10%, is used as food by humans and other animal species together. On the other hand, probably more than 10% of plants are used for medicinal purposes. A driving factor for the renewed interest in plant products in the past 20 years has been the rapid rate of plant species extinction as a consequence of tropical rain forests exploitation where more than half of the plant and animal species are found, as well as global warming. Consumers demand for “natural” products is also responsible for this renewed interest. It is estimated that the world is losing one major drug from these plants every two years [5]. Consequently, more of these compounds should be rapidly subjected to animal and human studies to determine their effectiveness in whole-organism systems, before they are lost. Plant extracts and plant-derived molecules concentrations tested *in vitro* could translate to unattainable high concentrations *in vivo*. Thus, some extracts or molecules could not be realistically considered for animal treatment.

Natural products derived from medicinal plants have proven to be an abundant source of biologically active compounds, many of which have been the basis for development of new lead chemicals for pharmaceutical companies. The safety of medicinal plants including herbals is of concern with all forms of these medicines. One health and safety issue concerns the potential interaction between plant extracts and prescribed drugs. The large number of pharmacologically active compounds in plant medicines increases the likelihood of interactions taking place. It is possible that a number of active molecules of plant extracts could act synergistically to produce the observed therapeutic effects [6]. Hence, isolating the components and using them individually could lead to a loss of the desired activity. These aspects have to be rigorously evaluated before a plant or a plant extract is declared safe and use on a large scale as therapeutic or prophylactic remedy.

## 2. Diarrhea and ETEC

Diarrhea is a clinical symptom marked by rapid and frequent passage of semisolid or liquid fecal material through the gastrointestinal tract. Secretory diarrhea occurs when an imbalance between absorption and secretion in the small intestine occurs. Various causes can explain this condition including virus, parasites and bacteria. Secretory diarrhea is predominantly a result of active secretion of chloride and bicarbonate ions. Secretion of these electrolytes leads to an osmosis-driven water movement into the intestines. Supportive care with replacement of intestinal fluid losses using oral rehydration solutions or isotonic intravenous fluids is the primary treatment [7]. Overall, decreasing the chloride secretion into the gastrointestinal tract presumably results in decreased stool weight and frequency, bringing symptomatic relief of diarrhea. 

ETEC is the most frequently isolated enteropathogen, accounting for approximately 200 million diarrhea episodes and about 380,000 deaths annually [8]. Gastrointestinal infections with enterotoxigenic *Escherichia coli* (ETEC) pose a major health problem among children younger than five years old in developing countries [9]. ETEC is also the leading cause of travellers’ diarrhea affecting annually over 10 million travellers to developing countries. Moreover, ETEC infections constitute a substantial problem in farm animals especially in newborn and early-weaned piglets [10]. Compared to other mechanisms of diarrheal disease, the hypersecretion related to ETEC enterotoxin activity is not associated with severe intestinal hitopathological changes [11].

## 3. Enterotoxins

**Figure 1:**
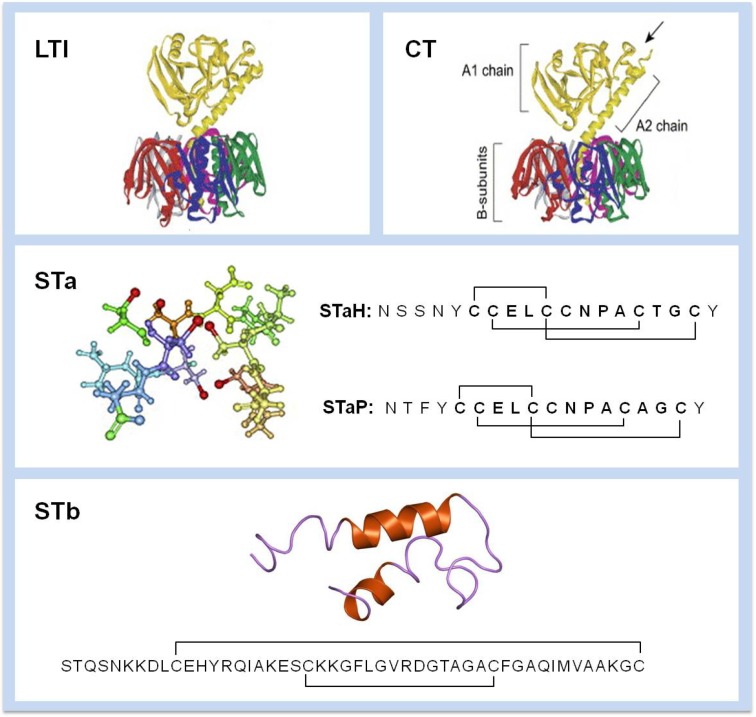
Structure of Enterotoxigenic *Escherichia coli* (ETEC) enterotoxins. LTI is related to *V. cholerae* CT toxin with the A subunit that is cleaved (site indicated by an arrow) into A1 and A2. The B subunits pentamer is responsible for receptor binding to GM1. The structures and sequences of STa (STaH and STaP) and STb are presented. Positions of disulfide bridges are indicated.

ETEC secrete 2 types of enterotoxins known as heat-labile (LT) and heat-stable enterotoxins (ST). Two types of LT exist: LT-I and LT-II. ETEC producing LT-I is usually associated with diarrhea in humans (LTh) and piglets (LTp) [12]. Both molecules are related to the cholera toxin (CT) of *Vibrio cholerae* but slightly differ antigenically from each other (Figure 1) [13]. ETEC can also produce two types of LT-II: LT-IIa and LTII-b. These toxins differ from LT-I and CT antigenically but possess similar toxic activity. These toxins can be produced by *E. coli* isolates from humans, bovine and water buffaloes [14]. The various LT toxins also differ in their main receptor specificity. LT-I (LTh and LTp) have GM1 as their main receptor, similar to CT. LT-II differs from LT-I as LT-IIa has GD1b and LT-IIb has GD1a as their main receptor [11]. 

There are two heat-stable toxins produced by ETEC: STa and STb. These toxins remain active after incubation at 100 °C for 30 min. STa are found as two types, STaH (19 amino-acids) and STaP (18 amino-acids) (Figure 1). These toxins are oligopeptide of *ca.* 2 kDa. STaH is only produced by human ETEC strains whereas STaP is produced by animal (porcine, bovine, ovine and canine) and human ETEC strains [15]. STb is a 48 amino-acid peptide of 5.2 kDa mostly associated with porcine ETEC strains [16]. Nevertheless, some ETEC isolates from human origin where shown to possess the gene for this enterotoxin [17,18]. 

### Relation between LT and CT

LT toxin is structurally and biologically related to CT (Figure 1). These enterotoxins share a similar mechanism of action by binding to the same receptor molecule (GM1) on the small intestinal epithelium and subsequently activating cyclic nucleotide second messenger. CT and LT stimulate the intracellular synthesis of cyclic adenosine monophosphate (cAMP). This compound initiates metabolic cascades characterized by net fluid and electrolytes secretions into the intestinal lumen. In the small intestine, epithelial Cl^−^ and carbonate secretion is activated and Na^+^ absorption is inhibited. 

Because LT-I and CT share an 83% amino acid sequence homology and the fact that LT causes a cholera-like diarrhea, plant products showing effects on CT will also be discussed in this review, as stated before [19]. LT is a major etiologic cause in traveler’s diarrhea, but as cholera is endemic to many regions of the world, it has been a major concern to the people inhabiting these areas and also to travelers to these parts of the world. Thus, cholera has been the object by local peoples of constant studies searching for ways to limit and/or control the germ and suppress the activity of the toxin responsible for the rice-water diarrhea observed. The importance of recurrent cholera epidemics has prompted researchers to look for products from indigenous plants to treat or at least diminish the symptoms of cholera. This can explain the high number of studies on CT compared to LT-induced secretory diarrhea.

## 4. Mechanism of Action

### 4.1. LT

**Figure 2:**
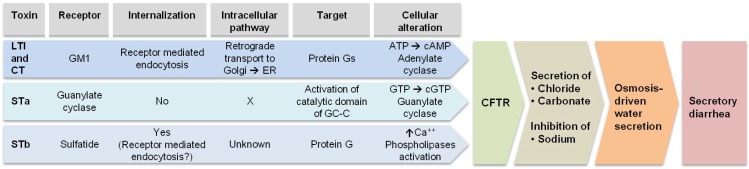
Mechanism of action of ETEC and *V. cholerae* enterotoxins leading to secretory diarrhea. Plant products can inhibit enterotoxins activity at different steps from toxin binding to receptor, uptake and the cellular alterations they provoke.

LT comprises one A enzymatically active subunit and five B subunits required for binding to its receptor (Figure 1). Diarrhea is induced by LT after binding of the B subunits (LTB) to GM1 ganglioside [Galβ1-3 GalNAcβ1-4(Neu5Acα2-3) Gal-β1-4 Glc-ceramide] present on the surface of intestinal epithelial cells. LT is then internalized through receptor-mediated endocytosis. Within endocytic vesicles and by retrograde transport it is translocated to the Golgi apparatus where it is disassembled. Then, the A subunit transits to the endoplasmic reticulum (ER). There, the A subunit is enzymatically cleaved to A1, a NAD-dependent ribosylating activity, and A2 fragment involved in linking the LTB subunits [20] (Figure 1). Inside the cytoplasm, the A1 subunit binds to ADP-ribosylating factors (ARF). The activated A1 subunit ADP-ribosylates the α subunit of a regulatory Gs protein [21]. An irreversible activation of the membrane bound adenylate cyclase results in production of cAMP [22]. The accumulation of this compound within the cell activates protein kinase A (PKA), which phosphorylates the cystic fibrosis transmembrane regulator (CFTR) [23]. Secretion of chloride and carbonate ions occurs and water follows through an osmosis-driven force. At the same time, PKA inhibits Na^+^ re-absorption by the Na^+^/H^+^-exchanger 3 through phosphorylation [11]. Altogether, these cellular changes lead to a watery cholera-like diarrhea (Figure 2).

### 4.2. STa

STa exerts its toxicity through activation of an intracellular signal cascade. The receptor for STa (STaH and STaP) is found on the tip of microvilli of the enterocytes. Guanlylate cyclase C (GC-C), a transmembrane glycoprotein, acts as the receptor [24]. STa binds to GC-C leading to the activation of its intracellular catalytic domain, which converts GTP to cyclic guanosine monophosphate (cGMP). Accumulation of this compound activates cGMP-dependent protein kinase II (cGMPKII). This kinase acts on the CFTR that is responsible for secretion of chloride and carbonate ions. Elevated cGMP also inhibits phosphodiesterase 3 (PDE3) that increases the cAMP level activating PKA. CFTR is phosphorylated by PKA and this enzyme also inhibits Na^+^ reabsorption by the Na^+^/H^+^-exchanger 3 (NHE3). Altogether, these changes lead to water secretion in the intestinal lumen as a result of an osmotically-driven process (Figure 2) [11].

### 4.3. STb

STb is produced by most porcine ETEC strains [16]. STb has no sequence or structural homology with STa toxin [16] (Figure 1). In fact, STb induces diarrhea without activating adenylate or guanylate cyclases [25]. This toxin contains two disulfide bonds required for toxicity expression. The molecule binds to galactose-3 sulfate moiety of sulfatide, an acidic glycosphingolipid present on intestinal epithelial cells [26]. STb has to be internalized to stimulate secretion [27]. Inside the cell, a G protein is stimulated resulting in calcium ions increase [28]. Elevated Ca^+^^+^ levels activate a calmodulin-dependent protein kinase II (CaMKII). This enzyme opens a calcium activated chloride channel. Activation of protein kinase C (PKC) occurs and CFTR is phosphorylated leading to HCO^3−^ and Cl^−^ secretion followed by an osmosis-driven water loss. PKC also inhibits Na^+^ uptake [11]. Phospholipases (A2 and C) provoke the release of arachidonic acid from membrane phospholipids and the formation of prostaglandin E2 and serotonin from enterochromaffin cells [29,30]. These mediators are also responsible for transport of electrolytes and water out of the intestinal cells (Figure 2).

## 5. Antibacterial Activities toward ETEC

Although 25%–50% of the current pharmaceuticals are derived from plants, none are presently used as antimicrobials [31,32]. Thus, screening for antibacterial activities may yield candidate compounds as potential new antimicrobial drugs. Over the last 20 years, numerous studies have been conducted on plants (using water and organic solvent extractions) including essential oils of what is known as medicinal plants. Although traditional medicine have associated certain plant products with healing of disease including gastrointestinal disorders, the mechanism accounting for antibacterial activities against enterobacteria which some are responsible for causing diarrhea are not thoroughly understood [1]. *In vitro* tests (disk diffusion and broth dilution methods) were conducted in search for effective treatment of diarrheal disease causing bacteria. In addition, plants with significant activity against enteropathogens could offer alternative methods to treat drug resistant enteric infections [33]. 

Plants have the capacity to synthesize an almost limitless number of aromatic compounds. A large proportion is constituted of phenols, or their oxygen-substituted derivatives [31]. Most of these compounds are secondary metabolites serving in many cases as plant defense mechanisms against predation by herbivores, insects, bacteria, fungi and viruses. Some of these substances are terpenoids responsible for the characteristic plant odors, other are plant pigments (quinones and tannins). Herbs and spices used by humans as food seasoning are also used as medicinal compounds as we will see later [34]. With increasing concerns over the use of in-feed antibiotics and metals, the weaning period of farm animals becomes more difficult to manage. There is thus a growing interest in natural products that could reduce enteric infections in animals.

Any kind of agent targeting bacterial viability can be expected to impose selective pressure on the development of antimicrobial resistance. In contrast, repression by natural compounds of bacterial virulence factors that do not affect bacterial growth has advantages such as preserving the host indigenous microflora with less selective pressure on the development of bacterial resistance [35,36,37]. Bacterial infections and the merging multidrug resistance are driving interest in fighting microorganisms with natural/plant products. Antibiotics kill bacteria but cannot inhibit the toxicity of the bacterial toxins that can be release following antibiotherapy thereby exacerbating the disease status. Moreover, antibiotic therapy is most probably not a viable solution because of the rapid increase in antibiotic resistance [35]. Today, the most common principle of management for diarrhea is the replacement of fluid losses (fluid replacement therapy) and/or the killing of the bacteria (antibiotics therapy).

**Figure 3:**
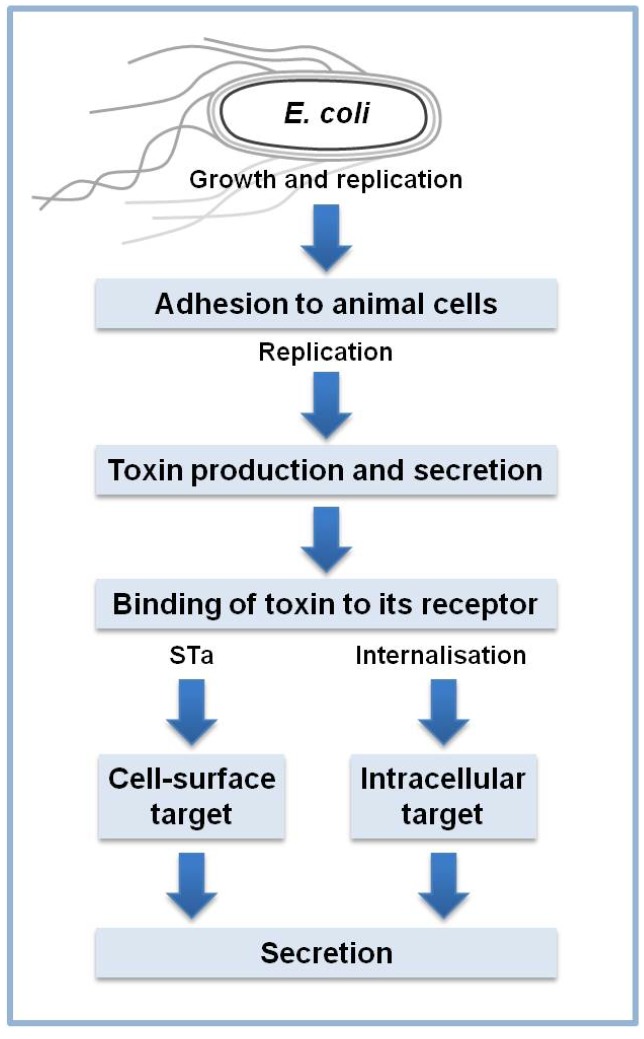
Pathogenesis steps for ETEC. Plant products can act at various levels, extracelluarly and intracellularly, to inhibit the action of the bacteria and/or enterotoxins.

Many plant products are known to inhibit the growth (bacteriostatic) or kill the bacteria (bactericidal) [31]. The same compounds at sub-bactericicidal concentrations can in some instances have a direct effect on ETEC enterotoxin without inhibiting the growth or killing the bacteria. The compounds could act at different levels of the pathogenic steps involved in secretory diarrhea induction (Figure 3). Antimicrobial activities of plant products based on bacterial targets unexploited by actual antibiotics could constitute a breakthrough for treating infections. This approach could be more relevant for treatment of etiological agents that are resistant to available drugs. In the best of cases, the plant products identified could be active only against pathogens without affecting the other microorganisms of the normal flora. Ultimately it would be most desirable to both specifically inhibit the growth of potential pathogens and/or increase the numbers of the gut microbiota that are beneficial to the animal (for example, Bifidobacteria in the intestine). The daily intake of active plant products might be expected to alter the growth and composition of microbial community. Various microorganisms are resident in the human intestinal tract and these participate in the normal physiological functions including the biotransformation of a variety of ingested or indigenously formed compounds to harmful derivatives. They can also be involved in the generation of beneficial products in a similar way as pre- and probiotics work [38]. Protection from a variety of disease can contribute to maintain optimal human health status. Many plant products have been studied for their antimicrobial activities either bacteriostatic or bactericidal. Experiments on the antibacterial activities of plant products are numerous and usually rely on *in vitro* tests [31]. 

Studies were conducted specifically on growth inhibition or killing of ETEC strains. Nevertheless, extracts and compounds able to inhibit *E. coli* strains of various pathotypes most probably have the same effect on ETEC. Many other papers related to growth inhibition and killing of *E. coli* strains have been published but these studies will not be described in detail in this review. Some examples of plant products often described in the literature for their antimicrobial properties toward *E. coli* and *V. cholerae* are described in the next chapter.

### Examples of Antibacterial Activities Related to Plant Products

The current knowledge obtained for some plant products and extracts with antibacterial activities (bacteriostatic or bactericidal) on ETEC (and *V. cholerae*) can be found in Table 1. 

**Table 1:** Plant products with antibacterial activities and/or active against ETEC and *V. cholerae* (CT) enterotoxins.

Plant	Scientific name	Specific compound	Target	Mechanism	Reference
Apple	*Malus spp*	Applephenon	*V. cholerae*	Inhibits CT ADP-ribosylation activity	[75]
Bean	*Vicia faba*	?	ETEC	Inhibits LT binding to GM_1_	[115,116]
Berberine*	*Berberis aristata*	Alkaloid	ETEC and *V. cholerae*	Effect on tight jonctions, NHE_3_ and AQP_4_ Inhibits secretory response of STa	[128]
Black tea*	*Camellia sinensis*	Catechins (EGCG)	ETEC	?	[69]
Cocoa	*Theobroma cacao*	Flavonoids	ETEC	Inhibits CFTR	[142]
Daio (kampo formulation)	*Rhei rhizoma*	Polygallate (rhubarb galloyl tannin)	*V. cholerae*	Inhibits CT ADP-ribosylation actvity	[76]
Elephant garlic	*Allium ampeloprasum*	Diallyl sulfides	*V. cholerae*	Growth inhibition	[39]
Fenugreek	*Trigonella foenum-graecum*	Galactomannans	ETEC and *V. cholerae*	Inhibits LT and CT binding to GM_1_	[148]
Flor de manita	*Chiranthodendron pentadactylon*	(−) Epicatechin	ETEC and *V. cholerae*	Interacts with CTA subunit	[144]
Flowering quince	*Chaenomeles speciosa*	Oleanic acid, ursolic acid, betulinic acid	ETEC	Inhibits LTB binding to GM_1_	[147]
Gall of R. sinensis*	*Galla sinensis*	Gallic acid (methyl gallate)	ETEC	Inhibits binding of LTB to GM_1_	[54]
Garlic	*Allium sativum*	Diallyl sulfides	*V. cholerae*	Growth inhibition	[39]
Ginger	*Zingiber officinale*	Zingerone and zingerol	ETEC	Inhibits LTB binding to GM_1_	[110]
Green tea*	*Camellia sinensis*	(−)-(Epigallocatechin-3-gallate), gallotanins	ETEC and *V. cholerae*	Inhibits calcium chloride channels	[73]
Guazyma	*Guazyma ulmifolia*	Procyanidins	*V. cholerae*	Interacts with CTA subunit	[146]
Hop	*Humulus lupulus*	Procyanidins	*V. cholerae*	Inhibits CT ADP-ribosylation activity	[74]
Kampo formulations	*Hange-Shashin-to* *(TJ-14), Keishi-Ka-Shakuyaku-to (TJ-60)*	?	ETEC	TJ-14 and TJ-60 suppress colon contractions TJ-60 blocks GCC activity (STa)	[77]
Liquorice	*Glycyrrhiza uralensis*	Glycyrrhizin (oleane-type triterpenoids)	ETEC	Inhibits LTB-GM1 interaction	[114]
Neem	*Azadirachta indica*	?	*V. cholerae*	Growth inhibition and antisecretory activity	[41]
Palmarosa	*Cymbopogon martinii*	Geraniol	ETEC	Growth inhibition	[32]
Pea	*Pisum sativum*	?	ETEC	Inhibits LT binding to GM1	[116,117]
Pepper*	*Piper longum*	Piperine	ETEC and *V. cholerae*	Antibacterial	[139]
Red chili*	*Capsicum annuum*	Capsaicin	*V. cholerae*	Inhibits CT production	[6]
Red seaweeds	*Gigartina sp.*	λ carragenin	ETEC	Mimicks STb receptor	[149]
Sangre de drago	*Croton lechleri*	Crofelemer	ETEC	Inhibits CFTR and calcium-activated chloride channels	[89]
Sumac*	*Rhus sinensis*	Gallotannins	ETEC	Antibacterial	[52]
Tempe	*Glycine max*	Arabinose-containing molecule	ETEC	Inhibits adhesion of F4	[47]
Wood creosote	*Fagus crenata*	Seirogan	ETEC	Antisecretory and antimotility (STa and LT)	[105]

Note: *: At higher concentrations these plant products showed antibacterial activity.

Using 29 medicinal plants commonly used in Brazil showed that ETEC strains of various serotypes were strongly inhibited by *Cymbopogon winteranius* [32]. A plant extract from *Aloysia triphilla* also showed moderate to strong growth inhibition. In that study, geraniol isolated from *Cymbopogon martini* was identified as an antibacterial compound. Considering that geraniol constitutes more than 60% of *C. martini* essential oil, results obtained suggested that it could be the main compound responsible for the antibacterial effect of the essential oil. Elephant garlic (*Allium ampeloprasum*) showed bactericidal activity on all strains of *V. cholerae* tested in relation to the content in diallyl sulfides was noted. This is not surprising, as Louis Pasteur himself had shown the antibacterial activity of garlic, *Allium sativum*. Garlic extract can also inhibit *V. cholerae* growth [39]. Pathogenic bacterial strains from Mexico (189 Gram-positive and 135 Gram-negative comprising 42 strains of *E. coli*) from pediatric patients severely infected were tested *in vitro* against 11 essential oils from commercial origin [40]. *Cinnannomum verum* (cinnamom), *Origanum vulgare* (oregano) and *Thymus vulgaris* (thyme) exhibited the highest and broadest antibacterial activity against the tested bacteria including *E. coli*. *Azadirachta indica* A. Juss (naliaceae) is known in India as *neem*. In that country, it is used for curing gastrointestinal disorders such as diarrhea and cholera. Methanol extract of neem leaf showed significant antibacterial activity against the multi-drug resistant *V. cholerae* of serotypes O1, O139 and non-O1, non-O139. Neem extract had an antisecretory activity of *V. cholerae*-induced fluid secretion in mouse intestine [41]. 

## 6. Tempe

Tempe (or tempeh) is a traditional fungal fermented food made from cooked soya beans. *Rhizopus microsporus* var. *oligosporus* is the microorganism traditionally used to produce tempe. *Bacillus* can also be used to ferment cooked soya beans [42]. A study by Kiers *et al.* showed that tempe could not inhibit *in vitro* growth or kill ETEC [43]. However, several tempe extracts were able to inhibit the adhesion of K88 (F4) ETEC to intestinal cells. Hence, this natural product could have potential as a feed supplement in the diet of weaned piglets against K88 ETEC infection. In addition to controlling diarrhea in ETEC challenged piglets, tempe significantly improved weight gain and feed intake [44]. Interestingly, *Rhizopus* fermented soya beans were more effective in controlling ETEC-induced diarrhea whereas *Bacillus* fermented product was promoting weight gain and feed intake more effectively. Another study by the same research group was conducted in ETEC-infected pig small intestinal segments and perfuse with tempe [45]. Tempe and cooked but not fermented soya beans showed minor fluid losses compared with saline control. Tempe resulted in high intestinal uptake of solutes in presence of an ETEC strain. Processed soya bean products particularly cooked soya beans and tempe were able to maintain fluid balance [45]. This indicated that tempe could be beneficial in the case of postweaning diarrhea in piglets and possibly in children as well. A subsequent study indicated that a high molecular weight soluble fraction (>5 kDa) of tempe was responsible for protection against fluid losses induced by ETEC [46].

Roubos van den Hill *et al.* (2009) confirmed the interference of tempe toward ETEC adhesion with a reduction of more than 80% for pig jejunal brush border cells and 50% to Caco-2 cells [47]. In this study, K88 ETEC bacteria were found to interact with soya bean extracts and this could be responsible for the observed decrease of ETEC adhesion to pig and human intestinal epithelial cells *in vitro*. This result strengthened previous observation of the anti-diarrheal effect of tempe. Again, none of the soya bean extracts inhibited bacterial growth and the extracts concentration tested in the study were physiologically relevant. In addition, the *in vivo* protective effect of tempe in piglets [44] implies that its bioactivity was not affected by digestive enzymes. 

Partial purification of tempe bioactive component showed its resistance to heating, defating and protease treatment. However, a treatment with a polysaccharide-degrading enzyme mixture abolished the activity. The active component was shown to be larger than 30 kDa with an increased carbohydrate content of higher arabinose [48]. This active component originates from the arabinan or arabinogalactan side chain of the pectic cell wall polysaccharides of the soya beans.

A recent study by Mo *et al.* (2011) comparing the anti-adhesive activity of tempe and tofu revealed that both have an anti-adhesive activity against *E. coli* *in vitro* [49]. Tofu had anti-adhesive activity although lower than tempe. After digestion through the stomach and the small intestine these product have even higher anti-*E. coli* adhesive activity confirming the potential antidiarrheal effect of tempe and most probably of tofu, as well. The active molecules could withstand the harsh pH in the stomach and pancreatic secretions and bile in the small intestine. Physiologically relevant concentrations (serving size of 100 g of soya) were tested. The *in vitro* anti-adhesion effect noted was dose-dependent. 

The interaction between soya beans and the bacteria resulted in a loss of adhesion capability of ETEC to the intestinal cells. The active component was either release or formed during the fermentation and was not present in the microbial mass and only partly in unfermented soya substrates. As a range of ETEC strains was sensitive to the bioactive component it makes tempe relevant for applications in animal husbandry, for example.

## 7. Sumac

*Rhus chinensis* belongs to the genus *Rhus* and the family *Anacardiceae* [50] and is commonly called sumac [51]. *Galla chinensis* or *Galla rhois* is the term used to describe the gall caused by the Chinese aphid, *Schlechtendalia chinensis* (Bell) on the leaves of *R. chinensis* [38]. This plant and its gall have a long history of use by indigenous people, especially in China, for medicinal care [52]. The galls are rich in gallotannin (50%–70%), a type of hydrolysable tannin. Gallotanins from *G. chinensis* consist of a central glucose core surrounded by several gallic acid units. Gall extracts was shown to inhibit the growth of several bacteria including *E. coli* with a minimal inhibitory concentration in the mg/ml range. Gallic acid and its derivative methyl gallate are the major components responsible for the antibacterial activity [53]. 

*G. chinensis* methanol extracts are also effective against ETEC induced diarrhea. In a mouse gut assay Chen *et al.* (2006) found that the extract of *G. chinensis* exhibited an anti-LT-induced diarrheal effect [54]. Competitive GM1-ELISA assay indicated that *G. chinensis* extract suppressed LT-induced fluid accumulation by blocking the binding of LTB to GM1. The active fraction was mainly composed of phenolic compounds especially gallic acid. Pure gallic acid significantly blocked binding of LTB to its receptor and could suppress fluid accumulation in a dose-dependent manner [54]. *In vitro* methyl gallate was also shown to possess good antibacterial activity against intestinal bacteria including *E. coli* [55]. Combinations of nalidixic acid and methyl gallate synergistically improved the inhibition of nalidixic acid resistant pathogens [55]. *In vitro* data suggested that combining nalidixic acid, methyl gallate, and carvacrol (*i.e.*, oregano essential oil) could be beneficial in controlling nalidixic acid resistant bacteria [55]. 

Other experiments done with the following *Rhus* plants indicated a role in diarrhea prevention. A methanol extract of *Rhus javanica* ripen fruits produce a significant reduction in the fecal output, and protected from the castor oil-induced diarrhea in Swiss albino mice [56]. In a study by Bose *et al.* (2008), a methanol extract of the fruits of *Rhus semialata* possesses significant antidiarrheal effect in rats and substantiated the use of this herbal remedy as a non-specific treatment of diarrhea in folk medicine [57]. The same effect was noted for *Rhus tripartitum* as shown by Abassi and Hani (2012) [57]. Traditional use of *R. tripartitum* for the treatment of diarrhea is attributable to the presence of antibacterial agents.

## 8. Tea and Tea Extracts

Tea leaves of *Camellia sinensis* produce organic compounds that may be involved in the defence of the plant against invading pathogens. These include insects, fungi, bacteria, and viruses. Chewing tea leaves is a common “folk remedy” in different countries suggested to be effective in ceasing diarrhea [58]. Tea is characterized by a high content of polyphenols called flavonoids. Polyphenols makes up approximately 30% of the dry weight of fresh tea leaf. Green tea contains mainly monomeric flavonoids whereas black teas contain mainly dimeric and polymeric flavonoids. Commercial teas are usually classified into three major categories: unfermented containing catechins, fully fermented black tea containing catechins, theaflavins, and polymeric thearubigins; and semifermented usually black oolong, containing both catechins and theaflavins. Tea composition (flavonoids content) and activities is influenced by growth environmental factors. The antibacterial activity of teas is related to the flavonoid levels which are also influenced by the degree of fermentation and the harvesting season [59]. Tea catechins can also protect against *V. cholerae* O1 infection [60].

Friedman M. *et al*. (2002, 2006, 2007) determined the antimicrobial effects of about 200 plant essential oils and their active components including tea components (catechins and theaflavins), and tea infusion [59,61,62]. Selected compounds active, *in vitro*, against non-resistant bacteria were also active against antibiotic resistant bacteria [59]. Physicochemical studies suggest that the antibacterial activities observed for galloylated tea catechins at the cell membrane level may be due to specific perturbations of the ordered structure of phosphatidylcholine and phosphatidylethanolamine bilayers of bacterial cell wall membranes [63,64]. (−)-Epigallocatechin-3-gallate (EGCG) was the most effective catechin causing leakage from *E. coli* membranes. EGCG, may be beneficial in colitis through selective immunomodulatory effects which may also be mediated, at least in part, by inhibition of NFkB and activator protein-1 [65]. A recent study by Verhelst *et al*. (2013) indicated that tea extract inhibited binding of LT to its GM1 receptor, as well as cAMP activity in Vero cells [66]. Polyphenols were shown to effectively induce formation of large (>100 kDa) of LT-polyphenol aggregates. Comparing seven polyphenols it was found that the most active molecule tested was pentagallaoylglucose suggesting that at least two galloyl moieties are required for the aggregating activity [67].

As stated before, interest in natural products and especially plant-derived compounds that could interfere with enteric infection is growing [68]. Bruins *et al*. (2006) studied the effect of different tea types and subfractions on the intestinal fluid and electrolyte losses involved in ETEC diarrhea [58]. Perfusion of ETEC-infected (LT^+^) jejunal segments of anaesthetized piglets perfused with either green tea extract or black tea extract (3 g/L) significantly inhibited fluid and electrolyte losses compared to control without tea extract. *In vitro* both black and green tea extracts delayed the exponential growth of ETEC (bacteriostatic activity) in a concentration-dependent manner. Nevertheless, in the pig model of diarrhea, none of the tea solutions inhibited ETEC either in the jejunal lumen or on the mucosa. In the perfusion model, the optimal inhibitory effect of black tea extract was achieved between 1.5 and 3.0 g/L corresponding to a normal strength tea infusion. Tea concentrations in the jejunum are expected quite similar to the concentrations of tea consumed. As black tea extract perfusion could compensate for the induced intestinal fluid loss induced by LT, inhibition of enterotoxin binding or inhibition of enterotoxin-induced fluid secretion may be part of the inhibitory effect of black tea. In fact, experiments in an Ussing chamber (a system used to measure the short-circuit current as an indicator of net ion transport across an epithelium) supported the claim that black tea extract is able to prevent cAMP-mediated Cl^−^ secretion. However, inhibition of Cl^−^ secretion could only be achieved when the extract was added 3 hours before but not when added 10 min. before forskolin addition (an adenylate cyclase activator) [58]. This indicates that tea had a prophylactic effect against LT but no therapeutic effect after diarrhea is initiated. This study showed that in piglet jejunal segments black tea extract of a normal tea infusion is capable to counteract ETEC-induced secretory diarrhea. 

Bruins *et al*. (2011) recently tested a black tea diet fed to piglets in order to evaluate the *in vivo* effect of black tea [69]. The animals were infected with an LT^+^-ETEC strain. Piglets fed with a 0.8% (wt/wt) black tea diet had the lowest diarrhea prevalence followed by those fed the 0.4% (wt/wt) and control diets, respectively. Similar reductions in diarrhea prevalence have been observed in calves fed green tea extracts [70,71]. Adverse effects of a black tea diet were seen as a decrease of feed intake, feed efficiency and weight gain. The pig aversion for black tea is likely caused by the astringent theaflavins [72]. One explanation for the antidiarrheal effect of black tea may be its capacity to bind iron thereby rendering this essential metal unavailable to ETEC and delaying its growth. In conclusion, black tea-containing diets decrease diarrhea prevalence by 20% over the total 27-day study period in post-weaning piglets *in vivo*. However, the reduced feed intake and growth performance from 13 days onwards is a major concern for application of such diet in animal herds.

More specifically, tannic acid where gallic or digallic acid substituents are present on a macromolecular scaffold (gallotanins), as present in green tea, inhibited calcium chloride channels (CaCCs) in multiple cell types but did not affect CFTR Cl^−^ channels [73]. Remarkably, a 100-fold dilution of a green tea infusion inhibited CaCCs by more than 50%. Tannic acid and green tea inhibited intestinal Cl^−^ secretion. Gallotanins are thus potent CaCC inhibitors providing a potential molecular basis for the antisecretory benefits of green tea. 

## 9. Plant Polyphenols, Rhubarb Galloyl-Tannin and Applephenon

Polyphenols are generally believed to have antibacterial activity due to their ability to precipitate proteins. Morianaga *et al*, (2005) have studied applephenon derived from immature apple, hop bract extract, Rhubarb galloyl (RG)-tannin, and Daio (*Rhei rhizoma*), respectively, for their activities against cholera, *in vitro* [74]. 

Hop bract extract and applephenon could inhibit binding of CT to Vero cells or to GM1 in a concentration-dependent manner [74]. RG-tannin (a heterologous polyphenol/gallate structure) did not show such activity but a high molecular weight fraction of hop bract extract inhibited CT ADP-ribosyl transferase activity. These observations were confirmed using Cy-3-labeled CT following toxin binding to cells. Thus, hop bract extract and applephenon were shown to interfere with toxin internalization. 

High molecular weight fraction of hop bract extract and applephenon could precipitate CT, CTA and CTB from solution by creating aggregates larger than 250 kDa. From these data, we can conclude that similar polyphenols can bind CT forming large aggregates on the cell surface inhibiting CT binding and/or internalization. In contrast, RG-tannin formed complexes with CT without interfering with its binding to Vero cells or GM1 and thus could not inhibit its internalization [74]. Studies have shown that plant polyphenols, RG-tannin, and applephenon act directly on CT by suppressing the ADP-ribosyl transferase activity and the induced fluid accumulation in mouse ileal loops [75,76]. Apple polyphenol extract (APE) inhibited CT-catalyzed ADP-ribosylation (of agmatine) in a dose-dependent manner [75]. It also diminished fluid accumulation in two diarrhea models in mice. In the sealed mouse model, even when given orally 10 min. after toxin injection, fluid accumulation was significantly inhibited. APE had a negative allosteric effects on CT-catalyzed NAD:agmatine ADP-ribosyltransferase. Fractionated APE on a Sephadex resin produced two fractions, which consist of highly polymerized catechin compounds, strongly inhibiting the ADP-ribosylation reaction. RG-tannin interfered with CT activity but not with that of STa enterotoxin [75]. Thus, polyphenols shows specificity in their interactions with bacterial toxins. Overall, the data suggested that the inhibitory effects of the polyphenols were not due to nonspecific binding to cells or to cell damage but from interaction with CT in solution and on the cell surface. The polyphenols used in the study were not cytotoxic at the concentration used [75].

## 10. Kampo

Kampo formulations are mixtures of traditional herbal and plant product medications used in China and Japan for many centuries to treat diseases such as cholera. In fact, based on their efficacy in some medical issues, kampo formulations were integrated in the National health system in Japan. *In vitro*, the kampo formulation Daio-Kanzo-to was shown to inhibit ADP-ribosylation and the elongation of Chinese ovary cells (CHO) [76]. In this formulation, the Daio component (*Rhei rhizoma*) is responsible for inhibiting CT activity. From *R. rhizoma* extract, rhubarb galloyl (RG) tannin, a compound with polygallate structure, was the most effective. Synthetic gallate analogues were shown to inhibit CT activities including ADP-ribosylation, elongation of CHO cells, and fluid accumulation in rabbit ileal loops. The inhibitory effect increased with the number of galloyl groups in the molecule. Gallate derivatives added after CT (7 G-M and 9 G-M) almost completely inhibited CT-induced fluid accumulation in the mouse ileal loop model. The concentration required was higher if added after CT. The effectiveness of the compounds when added after CT may be in relation to the time necessary for internalization and release of the catalytically active CTA subunit [76]. 

**Figure 4:**
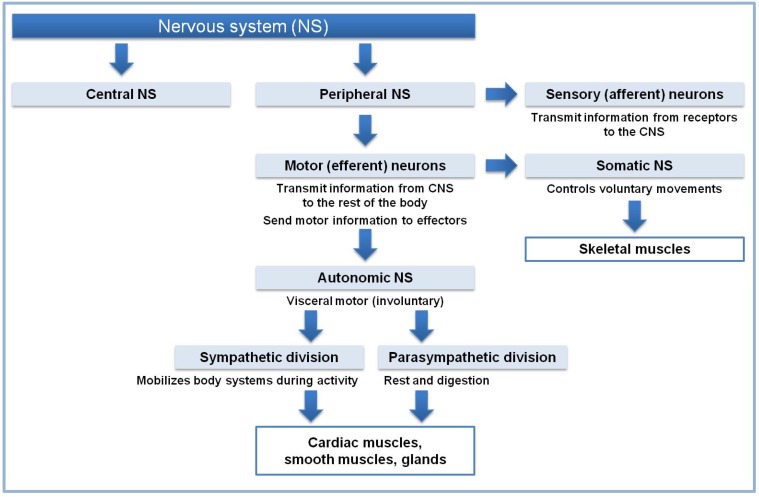
Functional organization of the nervous system. Bacterial toxins can act on this system at various levels.

Recently, Kito and Teramoto (2012) studied the Japanese Kampo, Hange-shashin-to (TJ-14) and Keishi-ka-shakuyaka-to (TJ-60), as traditional Japanese medicines used to treat human diarrhea on an empirical basis [77]. TJ-14 consists of seven herbs, namely *Pinelliae tuber*, *Scutellariae radix*, *Glycyrrhizae radix*, *Zizyphi fructus*, *Ginseng radix*, *Coptidis rhizome*, and *Zingiberis siccatum rhizoma*. TJ-60 consists of five herbs: *Cinnamomi cortez*, *Paeoniae radix*, *Zingiberis rhizoma*, *Zizyphi fructus*, and *Glycyrrhizae radix*. TJ-14 and TJ-60 at 1 mg/mL inhibited spontaneous contractions of circular smooth muscles of the rat distal colon by different mechanisms. The results suggested that TJ-14 and TJ-60 likely act through the production of nitric oxide (NO). Activation of small-conductance Ca^+^^+^-activated K^+^ channels seemed to be involved in the inhibitory effects of TJ-60. Since TJ-14 has inhibitory effects on myogenic and neurogenic contractile activity in the colon, it may well be effective in the treatment of abdominal pain associated with gastrointestinal motor dysfunction (Figure 4). 

Considering these results, Daio-Kanzo-to, RG-tannin, or other gallates (as discussed in Section 9) containing compounds could be added to oral rehydration solutions as an adjunctive therapy for the treatment of cholera patients in epidemic areas and possibly for traveler’s diarrhea. 

## 11. Sangre de Drago

The stem bark latex as well as the bark of the tree *Croton lechleri* is widely used by South American indigenous peoples for treatment of numerous ailments. Dragon’s blood (or Sangre de Drago) is used to describe the viscous red sap derived from several Croton species including *C. lechleri*, *C. dracanoides*, *C. palanostigma*, and *C. urucurana* [78]. The sap is obtained by slashing the bark of Croton and in general has low toxicity [78]. It is consumed orally (highly diluted 1:1000 or 1:10,000) for the treatment of severe gastrointestinal distress as a fast acting analgesic agent. This includes healing diarrhea and generalized cramping and discomfort. Saponins, sterols, alkaloids, proanthocyanidins, catechins, and diterpenes are present in the sap [79,80]. Dragon’s blood at an oral dose of 600 mg/kg caused a marked inhibition of diarrheal response following castor oil administration as well as the intestinal fluid accumulation promoted by CT suggesting potential usefulness of the red sap in the control of diarrhea [81]. In fact, dragon’s blood had a greater antisecretory effect compared to chlorpromazine a drug known for its effect in cholera patients. 

A study by Rao *et al*. (2007) indicated that the red sap had also an effect on pain [82]. A model of acute visceral pain in the mouse employing the intracolonic instillation of capsaicin or intraperitoneal cyclophosphamide showed that *Croton* red sap might mitigates visceral nociception. Additional cellular targets involved in intestinal ion transport was suspected as the crude extract from several *Croton* species could impair the capsaicin-stimulated ion transport across guinea pig ileum when added to the serosal side in Ussing chambers [83]. This effect was attributed to the ability of the red sap components to directly compromise sensory afferent nerves activation (Figure 4).

A compound termed SP-303 was isolated from crude Sangre de Drago obtained from *C. lechleri* [84]. This extract contains a purified, heterogeneous proanthocyanidin oligomer of which the basic monomers are (+)-gallocatechin, (−)-galloepicathechin, and to a smaller amount (+)-catechin and (−)-epicatechin. The oligomers consist of linearly linked monomers (5 to 11-mers with heptomers on average) of various ratios. This compound inhibited cAMP-mediated Cl^−^ and fluid secretion in T84 and Caco-2 cells as well as in mice intestinal loops treated with CT [85]. The safety and efficacy of orally administered SP-303 for symptomatic treatment of diarrhea was evaluated [86]. In a double-blind, randomized, placebo-controlled study among travelers to Jamaica and Mexico, SP-303 shortened the duration of acute secretory diarrhea by 21% without causing post-treatment constipation. SP-303 was safe and well-tolerated [87]. 

Evidence indicating that the major apically located Cl^−^ conductance in intestinal cells is mediated by the CFTR Cl^−^ channel has accumulated (Figure 2). Therefore, the CFTR Cl^−^ channel represents a primary drug target for blocking Cl^−^ secretion. In the laboratory, several molecules of different structural classes are used to block the CFTR but currently no drugs are available that specifically target and block this channel *in vivo*. Optimization of the manufacturing process of SP-303 resulted in a novel extract termed SB-300 containing 70% SP-303 by weight [88]. This compound is commercially available as a dietary supplement (NSF/Normal Stool Formula^TM^, Shaman Pharmaceuticals, Inc., San Francisco, CA, USA). 

SB-300 and SP-303 were shown to be direct blockers of CFTR Cl^−^ conductance activity from the extracellular side. Since these compounds are high molecular weight (3000 Da for SB-300 and 2200 Da for SP-303), transport across the membrane at a sufficient rate to reach a potential intracellular target seemed unlikely. In addition, when given orally, SP-303 was not detected in the serum of patients indicating that it could not permeate the intestinal wall [87]. Yet, these compounds are effective at low micromolar range and could be used as broad-spectrum antidiarrheal agent [85]. Thus, SB-300 could serve as a complementary and alternative approach for the treatment of pediatric diarrhea especially in the developing world where cheap drugs are more than welcome. 

Crofelemer is a trade name of a drug that is a purified oligomeric proanthocyanidin from *C. lechleri* obtained by distillation of Sangre de Drago. Crofelemer is a complex mixture of procyanidins and prodelphinidins with up to 30 (epi)catechin or (epi)gallocatechin units per molecule, resulting in a molecular mass of up to 9000 Da [89,90]. This compound is an antidiarrheal agent that simultaneously targets two distinct channels, the CFTR and the calcium-activated chloride channel. This product inhibits cAMP-stimulated CFTR chloride channels found on the apical membranes and CaCCs found on the epithelial membranes in the intestine [7]. Oral Crofelemer is a non-absorbed agent [85] that was well tolerated in clinical studies. No other agents on the market specifically target the CFTR and CaCC chloride channels for secretory diarrhea. The study by Bardhan *et al*. (2009) suggested that Crofelemer may improve diarrhea symptoms, especially in those patients with diarrhea caused by ETEC [91]. While antimicrobial agents should be utilized when indicated, Crofelemer may be beneficial in addition to fluid rehydrating therapy. It could reduce symptoms of diarrhea and also pain in patients. In traveler’s diarrhea, it provides a non-antibacterial treatment strategy with mild, non-bloody diarrhea that could theoretically decrease the use of antibiotics and subsequent sequelae of antibiotic resistance.

## 12. Wood Creosote

Wood creosote, an oily liquid, is a beech wood extract composed of guaicol, creosol and other related phenolic compounds which are widely used as an anti-diarrheal agent in Asia. In Japan, Seirogan, is a commercially available wood creosote preparation used as antidiarrheal medicine. Using rat jejunum and colon in Ussing chambers, transmural potential difference was used as an electrical marker of changes in mucosal ion transport [92]. The anti-diarrheal effects of Seirogan occur through a decrease in basal transmural potential difference in the upper small intestine and an inhibition of active secretion in the jejunum, ileum and colon. Seirogan-induced changes in ion transport have a non-neuronal mechanism of action. In fact, as experiments were performed on muscle-stripped intestinal preparation, the changes in mucosal ion transport could not be dependent on either smooth muscle activity or mucosal blood flow. Wood creosote showed equal potency in both jejunum and colon. The antidiarrheal action of wood creosote is due, at least in part, to its antisecretory activity as revealed in Ussing chambers using rat jejunum and colon epithelia [93]. A proabsorptive effect could also be involved in the beneficial activity observed toward diarrhea. 

Ataka *et al*. (1996) showed that local administration of wood creosote together with a fixe dose of LT suppressed LT-induced fluid secretion in a dose-dependent manner in ligated rabbit jejunum [94]. Kuge *et al*. (2001) using Ussing chambers with rat jejunum and colon and LT and STa toxins observed an antisecretory activity in the small intestine, against LT-induced secretion [95]. The secretion inhibition was more potent following serosal application whereas in the colon it inhibited STa-induced secretion with equal potency following serosal or mucosal addition. Wood creosote suppressed STa-induced increase in Isc (short circuit current) in a concentration-dependent manner, without causing a complete inhibition [96,97]. STa-induced fluid secretion in ligated rabbit jejunum was also inhibited. At the same time the spontaneous phasic, acetylcholine-induced tonic contractions, of the longitudinal and circular muscles of ileum was inhibited in a dose-dependent manner. This indicated that wood creosote had an antisecretory and antimotility effects toward STa toxin.

Seirogan inhibits intestinal secretion and normalizes the transport of electrolytes and water in rats subjected to restrain stress [97,98]. Epithelial transport was studied in modified Ussing chambers using isolated rat jejunum and colon. Wood creosote was able to reduce the stress-induced increase in Isc, electrical conductance and the mucosal to serosal flux of horse radish peroxidase compared to the vehicle-treated groups. In F-344 rats, oral administration of wood creosote prevented stress-induced diarrhea by impeding aversive effects on small intestinal secretion and barrier function [98]. 

The therapeutic effect of wood creosote was first linked to its ability to reduce intestinal motility [99,100]. Wood creosote was also shown to prevent the increase in colonic motility induced by corticotrophin-releasing factor through 5-HT(3) receptor in the proximal colon and through 5-HT(4) receptors in the distal colon [101]. In Yucatan mini-pigs Seirogan reversed stool softening produced by bisacodyl (a stimulant laxative drug acting on the colon to produce bowel movement) and restore the motility index by reducing the number of contractions [102]. Thus, inhibition of proximal colonic motility may contribute to the antidiarrheal action of Seirogan. 

Overall, the antisecretory effect is independent of the intracellular pathway activated by the enterotoxin since wood creosote inhibited both cAMP- and cGMP-mediated secretion induced by LT or STa, respectively [95]. Wood creosote may diminish the secretion induced by the interaction of bacterial enterotoxin with both epithelial cells and enteric neurons. The results showed the efficacy of wood creosote throughout the entire intestinal tract and wood creosote had no effects on normal intestinal activity [103,104]. The safety, tolerability and pharmacokinetics of a single dose of wood creosote tested in a randomized, double-blinded placebo controlled study in 40 human volunteers (14–42 years) indicated that single oral dose were safe and well tolerated in healthy men and women [103]. Creosote was rapidly absorbed, conjugated and eliminated. Wood creosote was more efficacious in relaxing abdominal pain and was comparable to loperamide (the available remedy) in relieving diarrhea. The antidiarrheal effects of wood creosote are due to both antisecretory activity in the intestine and antimotility in the colon but not to the microbiocidal activity as previously thought [105].

## 13. Red Chili

Red chilli, the fruit of *Capsicum annuum*, is a common pungent spice. One of its active pharmaceutical ingredients is capsaicin (N-anillyl-8-methyl-nonenamide). A methanol extract of red chilli, containing capsaicin inhibited CT production in *V. cholerae* El Tor variant strains without affecting its viability [6]. Capsaicin drastically inhibited CT production in *V. cholerae* strains of various serogroups including variants. Real-time quantitative reverse transcription-PCR assays indicated that capsaicin effectively repressed the transcription of virulence genes *ctxAB* (encoding CT), *tcpA* (encoding the toxin-coregulated pili TCP), and *toxT* (regulating the expression of CT and TCP) genes, but not of *toxR* and *toxS* genes that are responsible for the activation of the expression of ToxT [106,107]. On the other hand, capsaicin significantly enhanced the transcription of the *hns* gene, the product of which is known to regulate negatively the transcription of *ctxAB*, *tcpA* and *toxT* genes [108]. Thus, capsaicin might act as a potent repressor of CT production possibly by enhancing the transcription of *hns* coding for a histone-like nucleoid structuring protein (HNS). 

In fact, sub-bactericidal concentrations (100 μg/mL) of red chilli methanol extract could drastically inhibit CT production (≥90%) in 4 *V. cholerae* El Tor variant strains [6]. Red chili contains compound(s) that can inhibit CT production in *V. cholerae* regardless of their serogroups and biotypes. The inhibitory effect of capsaicin appears to be a general phenomenon and not strain specific. Also, red chilli extract showed higher inhibitory impact in comparison to capsaicin which indicates possibly that other unidentified compound(s) in red chilli that can directly inhibit or synergistically act with capsaicin. 

## 14. Ginger

Ginger, the rhizome of *Zingiber officinale*, is a traditional medicine used for the treatment of gastrointestinal diseases, including diarrhoea. Borelli *et al.* (2004) evaluated the effect of ginger on electrostimulation or acetylcholine induced contraction in isolated rat ileum [109]. Ginger inhibited both type of contractions being more potent in inhibiting the electrostimulated contractions. Using specific receptor antagonists. it was demonstrated that ginger had inhibitory effects on prejunctional and postjunctional ileal contractility. 

A methanol extract of ginger significantly blocked the binding of LT to cell-surface receptor GM1, resulting in the inhibition of fluid accumulation in ileal loops in mice [110]. Zingerone (vanillylacetone) is likely the active component responsible for the antidiarrheal activity. Chemically synthesized zingerone derivatives revealed that one of these molecules (2-[(4-methoxybenzyl)] benzoic acid) significantly suppressed LT-induced diarrhea in mice due to an excellent surface complimentarity with the B subunits of LT. This compound was more active than zingerone both *in vitro* and *in vivo*. The compound interacted with LTB via hydrogen bonds and hydrophobic contacts. 

Confirming the results of Borelli *et al.* (2004) [109] and Iwami *et al.* (2011) [111] showed that intraluminal application of zingerone, a pungent component of ginger, inhibited spontaneous contractile movements in isolated rat colonic segments in a dose-dependent manner. *In vivo*, zingerone also attenuated colonic motility without affecting blood pressure and heart rate. The effects on colonic movements were not affected by pretreatment with capsazepine or tetrodotoxin that blocks neural components. Thus, this compound can inhibit colonic motility through direct action on smooth muscles (Figure 4). The effects were reversible and reproducible. As the pungency is undesirable in therapeutic use, an analogue of zingerone, zingerol was tested afterwards [112]. This compound had no inhibitory effect on the jejunum, inhibiting the contractile movements in isolated colonic segments only, suggesting it was directly acting on the colon. Ginger has no side effects and no known drug interactions. 

## 15. Licorice

In several ancient cultures, *Glycyrrhiza uralensis* has been used for treatment of gastrointestinal disorders, such as diarrhea. Licorice root has been used in Europe since prehistoric times. Its use is well documented starting with the ancient Greeks [113]. It is also largely used as flavoring and sweetening agent. Glycyrrhizin, the principal component of licorice is a glycoside occurring as a mixture of calcium, sodium and potassium salts of glycyrrhizinic acid (also known as glycyrrhic acid). Chen *et al.* (2009) studied the effect of inhibitory traditional medicinal herbs (28 families) on LTB-GM1 interaction using an ELISA test [114]. Likely active phytochemicals of traditional medicinal herbs were then predicted by *in silico* model (docking technology concerns the study of 3D interaction of phytochemicals with LTB) and analyzed *in vitro* (GM1-ELISA) and *in vivo* (mouse gut assay) models. Docking data showed triterpenoids were the most active phytochemicals and the oleanane-type triterpenoids presented the best LTB-binding abilities. In *in vitro* and *in vivo* models, glycyrrhizin was the most effective oleanane-type triterpenoids that significantly suppressed both the LTB-binding ability and the LT-induced fluid accumulation in mice. Glycyrrhizin exhibited the lowest binding energy score among the oleanane-type triterpenoids. *G. uralensis* extract and glycyrrhizin reduced the binding of LTB to GM1 by 73.3% and 97.5%, respectively. Glycyrrhizin was capable of suppressing the LT-induced diarrhea at 10 mM. *In vitro* and *in vivo* models clearly showed that glycyrrhizin inhibited LT-induced diarrhea via the inhibition of GM1 and LTB interaction [114]. 

## 16. Pea and Fava Bean Hulls

Pea (*Pisum sativum*) and bean (*Vicia faba*) hulls were compared, *in vitro*, for interference with LT activity. Pea hull and meal fractions showed a higher binding of ETEC (K88ac) than faba beans [115]. In contrast, bean hulls proved more effective than pea hulls in preventing GM1 receptor binding of LT. The results suggested that fava bean hulls, contrary to the pea hulls, interfere with LTp-1 binding to GM1 in the intestine rather than having an effect on ETEC cell adhesion [116,117].

## 17. Berberine

Berberine is a plant alkaloid with a long history of medicinal use in both Ayurvedic and chinese medicine. It is present in many plants including *Hydrastis canadensis*, *Coptis chinensis*, *Berberis aquifolium*, *Berberis vulgaris*, *Berberis aristata*, and *Rhizoma coptitis*. The berberine alkaloid can be found in the roots, rhizomes, and stem bark of the plants. Diarrhea cause by *V. cholerae* and *E. coli* has been the focus of numerous berberine studies and results indicate several mechanisms, which may explain its ability to inhibit bacterial diarrhea. As early as 1982, berberine was shown, *in vitro*, to directly inhibit *V. cholerae* and *E. coli* enterotoxins effects [118]. In the case of *E. coli*, *in vitro* research indicated that berberine sulphate was capable of inhibiting bacterial adherence to the mucosal or epithelial surfaces as fimbrial structure formation was suppressed by this compound [119].

Berberine derived from the roots and bark of *Berberis aristata* inhibited by approximately 70% the secretory responses of CT and LT in the rabbit ligated intestinal loop model [118]. The drug was effective when given either before or after enterotoxin binding and when given either intraluminally or parentally. It did not inhibit the stimulation of adenylate cyclase by CT. Berberine also markedly inhibited the secretory response of STa in the infant mouse model. This compound possibly acts at a biochemical step after cyclase activation or it may be acting nonspecifically to enhance intestinal absorption. 

Ethanol and aqueous extracts of *B. aristata* bark delayed and reduced the number of diarrheal episodes in Swiss albino mice in a dose-dependent manner [120]. Minimal inhibitory concentration and minimal bactericidal concentration of berberine were almost comparable to ciprofloxacin. This study validated *in vivo* and *in vitro* antidiarrheal activity of *B. aristata* extracts. Bandyopadhyay *et al.* (2013) showed that the antibacterial activity is attributable to the strong binding of berberine to nucleic acids [121], corroborating previous studies [122,123]. Interestingly, *in vitro* antibacterial activity was noted against multidrug resistant ETEC strains isolated from yaks [121].

Gu *et al.* (2009) investigated the effect of berberine on tight junctions (TJ) in Caco-2 cell line [124]. Epithelial gut permeability as determined by transepithelial resistance (TER) and TJ morphology was reduced. The study showed that berberine could reinforce TJs and show a dose-dependent effect in the range of 25 to 100 mM after 4 h incubation. The effect was reversible. It also indicated that berberine is nontoxic to human enteric epithelium cell line. As berberine has low bioavailability and shows poor adsorption through the gut wall (<5%) [125], it supports the thesis that this compound may exert its antidiarrheal effect in intestinal epithelial cells before its absorption [126]. 

TNFα-induced barrier defect in HT-29/B6 human colon monolayers as revealed in Ussing chambers was prevented by berberine [127]. This effect was confirmed in rat colon. Berberin prevented TNFα-induced claudin 1 dissasembly and upregulation of claudin 2. This effect was mediated via tyrosine kinase, pAKT and NFkB pathways. As the effect of berberine relies on a novel mechanism, it suggests a potential therapeutic approach against barrier breakdown in intestinal inflammation. 

In order to understand the effect of berberine on ion exchange and water transfer, the expression of Na^+^/H^+^ exchanger 3 (NHE3) and aquaporin 4 (AQP4) in a diarrhea mouse model and in human intestinal epithelium cell line (HIEC) was evaluated [128]. The expression levels of NHE3 and AQP4 were significantly increased in the diarrheal mice treated with berberine compared to untreated mice. The same effect was observed in HIEC. No significant change of PKC activity was observed in the different HIEC treated groups. Although the maximal absorption of berberine was approximately 0.01%, berberine was able to increase the expression of NHE3 and AQP4 suggesting this compound might exhibit its antidiarrhea effect by enhancing the absorption of Na^+^ and water. 

Overall, the recent studies indicate that berberine has various pharmacological effects including inhibition of intestinal fluid accumulation and ion secretion [129], anti-inflammatory [130], antimicrobial [131], anti-inhibition of smooth muscle contraction [132], and action on TJs [124] each of which may contribute to the antidiarrhea effect. The therapeutic dosage for most clinical usage is 200 mg orally two to four times daily [133]. 

## 18. Piperine

The black and long pepper fruits of *Piper nigrum* and *Piper longum*, respectively, contain a large number of alkaloids and related compounds, the most abundant of which is piperine [134]. Piperine (1-piperrylpiperidine) was shown to inhibit the gastric emptying of solids/liquids in rat and gastrointestinal transit of mice in a dose-dependent manner [135,136,137]. The effect is local, concentration-dependent, and not centrally mediated. Its effect was however less potent than loperamide, the control drug. It acts as a gastrointestinal motility inhibitor and has also a probable effect on prostaglandins. No effect on permeability of human epithelial cell monolayers *in vitro* was noted. Petrol ether and ethyl acetate extracts of *P. Longum* were found to exert antimicrobial effects against various microorganisms [134,138]. 

Pikutbenjakul is a Thai medicinal plant formula containing *P. longum*, *Piper sarmentosum*, *Plumbago indica* and *Zingiber officinale*. *In vivo*, a 95% ethanol extract of this preparation showed antibacterial activities against ETEC and *V. cholerae* [139]. Crude pepper extract and piperine showed the presence of spasmodic (choligernic) and antispasmodic (opioid agonist and Ca^+^^+^ antagonists) effects [140].

## 19. Cocoa

Reports dating back to the 15th century indicate traditional cocoa preparations were used by indigenous people of Central America to treat childhood diarrhea and other intestinal ailments [141]. Cocoa is a rich source of polyphenols consisting largely of oligomeric procyanidins ranging from mono- to decamers. Flavonoids compounds of cocoa tested on forskolin-stimulated CFTR-mediated Cl^−^ secretion (mimicking LT- and CT-induced secretion) across T84 colonic epithelial cells in Ussing chambers indicate that these compounds act as mild CFTR blockers [142]. It was suggested that normal cocoa consumption results in sufficient concentrations to affect intestinal CFTR-mediated salt and water secretion by the small intestine. As flavonoids are poorly absorbed by the human intestine, it results in quite high concentrations in the intestinal lumen. 

## 20. “Flor de Manita” or Macpaxochitl

The flowers of *Chiranthodendron pentadactylon* Larreat, “flor de manita”, have been traditionally used as folk medicine in Mexico [143]. Methanol extract and subsequent fractions of flavanoids evaluated on CT-induced intestinal secretion in rat jejunal loops model revealed an antisecretory activity [144]. Epicatechin, present in the extract, exhibited the most potent antisecretory activity close to that of loperamide, the drug used as control. The ethyl acetate soluble fraction obtained from the plant crude extract, from which (−)-epicatechin was isolated, showed the best inhibitory activity (88.2% inhibition). The results obtained in the study supported the anecdotal report for the traditional use of the flowers of this plant in the control of dysentery. 

The antisecretory activity of 26 medicinal plants used in Mexico was tested in cholera-induced intestinal secretion in rat jejuna loop model. Certain methanolic and aqueous extracts at 300 mg/Kg were active against intestinal secretion response to CT. From the tested plants, *Chiranthodendron pentadactylon*, *Hippocratea excelsa*, *Ocimum basilicum*, *Geranium mexicanum* and *Bocconia frutescens* were the most active with inhibition values ranging from 68% to 93.4%. In general, the methanolic extracts exhibited the highest antisecretory activity [145]. 

These results are in agreement and could explain the results previously obtained by Hör *et al.* (1995) with antisecretory oligomeric proanthocyanidins of the plant Guazyma (*Guazyma ulmifolia*) which monomeric unit are (+)-catechin and (−)-epicatechin [146]. Rabbit distal colon mounted in Ussing chamber showed that an extract from the bark of *G. ulmifolia* interacted with CTA subunit. The most active fraction contained procyanidins with a polymerization higher than 8. The extract was active if added prior to the toxin to the mucosal side but had no effect if added after CT [146]. 

The ethyl acetate extract of *C. pentadactylon* tested against CT or STa toxin-induced intestinal secretion in rat jejunal loops models at oral doses of 10 mg/Kg exhibited the most potent effect on CT (59.9% inhibition) [144]. For STa toxin, the effect was moderate (24.1% inhibition). Computational molecular docking showed that epicatechin interacted with four amino acid residues (Asn103, Phe31, Phe223 and Thr78) found in the catalytic site of CT (CTA). This observation reveals a potential binding mode at the molecular level confirming the potential of epicatechin found in “flor de manita”, as a new antisecretory compound. 

## 21. Flowering Quince or *Chanomeles*

The fruit of *Chaenomeles speciosa* has been used to treat diarrhea in China. A study by Chen *et al.* (2007) showed that an extract of these fruits inhibited LT-induced diarrhea in mice by blocking the binding of LTB to GM1 [147]. The ethyl acetate soluble fraction was the most active fraction. Furthermore, oleanolic acid, ursolic acid, and betulinic acid from ethyl acetate fraction, blocked the toxin binding effects resulting in suppression of LT-induced diarrhea. In conclusion, the results suggested that these compounds were the active constituents. Therefore, they might be used as potent inhibitors for the treatment of LT-induced diarrhea.

## 22. Fenugreek

The interfering potential of different substances against CT and LT toxins were evaluated, *in vitro*, by Becker *et al.* (2010). When supplied before CT- and LT-GM1 complex formation, ground fenugreek seed (*Trigonella foenum-graecum*) counteracted GM1 binding to LTh-1 as well as to LTp-1 (43%–65% inhibition) and CT (61%–92% inhibition) [148]. With 50 mg/mL fenugreek seed, inhibition of even 92%–99% was reached for LTh-1 and CT binding. The study suggested that galactomannans might be the active principle of fenugreek. Efforts to resolve already bound toxin from GM1 with test substances were less effective than preincubations and concurrent incubations. Tara gum was the most potent of all substances tested in the after-treatment and this compound might offer some potential to resolve bound LTp-1 from GM1, but not LTh-1 or CT.

## 23. Algae

Algae are not plant per se but they belong to the vegetal kingdom. Most are photosynthetic but lack many distinct cell and organ types found in land plants. The largest and most complex marine forms are called seaweeds. Carragenins are a family of linear sulfated polysaccharides that are extracted from red seaweeds. All carragenins are high-molecular-weight polysaccharides made up of repeating galactose units and 3,6 anhydrogalactose (3,6-AG), both sulfated and nonsulfated. The units are joined by alternating alpha 1–3 and beta 1–4 glycosidic linkages. Many types exist and they have the ability to form a variety of different gels at room temperature. They are widely used in the food industry for their gelling and thickening properties. λ type carragenins are peculiar as it does not form a gel but remains soluble in solution. This compound was shown to inhibit the binding of STb toxin to its receptor, sulfatide, using surface Plasmon resonance technology [149]. In parallel, it was also shown to prevent STb internalization in IPEC-J2 (pig jejunal cells). As some galactose residues from λ carragenin are sulfated in position 3, as found in sulfatide, it could explain the mimicking of STb receptor and the competition observed. This compound is not toxic for animals and thus it could probably be added to pig feed as a prophylactic agent to prevent STb-induced secretory diarrhea. 

## 24. Conclusions

From the conducted studies, we can conclude that some plant products use in traditional or alternative medicines can represent active treatments against ETEC enterotoxins and CT-induced diarrhea. For some of the plant constituents, mainly tannins and similar compounds, the exact nature of the toxin inhibitory blockade was elucidated. Cocktails comprising the identified active compounds from different plants could be envisioned as a possible way to increase the inhibition of enterotoxin activity. This could be more relevant if these compounds are known to act at different steps of toxin interaction with the target cells. When considered for treatment, the concentration of these compounds should be below the toxicity level. For some compounds, this requirement could be unattainable.

It is amazing to realize that plants used for curing diseases have been selected through trial and error process over thousands of years of human experimentation. Nevertheless, we should also not minimize the toxicity associated with some plant and plant products. This will have to be accounted for when designing new drugs. In addition, the interaction of these new products should not or interact minimally with prescription drugs that are increasingly used, on a daily basis, by a large part of the world’s population. 
